# microRNA profilings identify plasma biomarkers and targets associated with pediatric epilepsy patients

**DOI:** 10.1038/s41390-023-02864-z

**Published:** 2023-10-27

**Authors:** Qi Wang, Xulai Shi, Ping-Ping Li, Li Gao, Yueyuan Zhou, Luyao Li, Hao Ye, Xiaoqin Fu, Peijun Li

**Affiliations:** 1 Department of Neonatology, The Second Affiliated Hospital and Yuying Children’s Hospital, Wenzhou Medical University, 325000 Wenzhou, Zhejiang China; 2 School of life Science and Biotechnology, Shanghai Jiao Tong University, 200240 Shanghai, China; 3 Key Laboratory of Structural Malformations in Children of Zhejiang Province, 325000 Wenzhou, Zhejiang Province China; 4 Key Laboratory of Pediatric Anesthesiology, Ministry of Education, Wenzhou Medical University, Wenzhou, China

## Abstract

**Background:**

Although previous studies show that microRNAs (miRNAs) can potentially be used as diagnostic markers for epilepsy, there are very few analyses of pediatric epilepsy patients.

**Methods:**

miRNA profiles using miRNA-seq was performed on plasma samples from 14 pediatric epileptic patients and 14 healthy children. miRNA miR-27a-3p that were significantly changed between two groups were further evaluated. The potential target genes of miR-27a-3p were screened through unbiased mRNA-seq and further validated using Western blot and immunohistochemistry in HEK-293T cells and in the brains of mice with epilepsy induced by lithium chloride–pilocarpine.

**Results:**

We found 82 upregulated and 76 downregulated miRNAs in the plasma from pediatric patients compared with controls (*p* < 0.01), of which miR-27a-3p exhibited a very low *p* value (*p* < 0.0001) and validated in additional plasma samples. Two genes, GOLM1 and LIMK1, whose mRNA levels were decreased (*p* < 0.001) with the increase of miR-27a-3p were further validated in both HEK-293T cells and in epileptic mice.

**Conclusions:**

MiR-27a-3p exhibits potential as a diagnostic and therapeutic marker for epilepsy. We postulate that additional studies on the downstream targets of miR-27a-3p will unravel its roles in epileptogenesis or disease progression.

**Impact:**

- A total of 158 differentially expressed miRNAs were detected in plasma between epileptic and control children.
- Plasma miR-27a-3p was one of the miRNAs with a low *p* value.
- GOLM1 and LIMK1 were validated as downstream target genes of miR-27a-3p.
- miR-27a-3p has potential as a diagnostic and therapeutic marker for epilepsy.

## Introduction

Epilepsy is a common nervous system disorder in childhood and affect 0.5–1% of children with the highest incidence in infancy.^1^ Repeated seizures cause brain cell hypoxia, apoptosis, neuronal loss, and abnormal synaptic reorganization that result in varying degrees of developmental delay, learning and behavioral difficulties, and intellectual disability. Although there are many treatment options—including antiepileptic drugs, surgery, and dietary interventions—up to 30% of epilepsies in children are medication-resistant.^2^ Because the underlying mechanisms of epileptogenesis are not fully elucidated, a better understanding of the underlying biological process is needed to find new treatment strategies and therapeutics. Finding reliable molecular biomarkers for epilepsy will also considerably guarantee an earlier diagnosis and timely treatment for pediatric patients.

MicroRNAs (miRNAs) are candidate biomarkers for diagnosis in epilepsy and potential therapeutic targets in the epileptic brain.^3^ MiRNAs are small (20–23 nucleotides) non-coding RNAs that post-transcriptionally regulate the expression of a target gene and act primarily by identifying partially complementary ribonucleotide sequences in the 3′-untranslated region (3’UTR) of the mRNA of a target gene. Studies have shown changes in miRNA levels of brain tissues and blood samples taken from epileptic patients and epileptic animal models.^4^ MiRNA-mediated mechanisms in epilepsy are recognized to contribute to multiple biological processes such as apoptosis,^5^ neuroinflammation,^6^ autophagy,^7^ and oxidative stress.^8^ Furthermore, inhibition of epilepsy-related miRNAs can reduce the onset of spontaneous epilepsy^9^ and may have potential as a diagnostic marker for epilepsy. Unlike mRNA, miRNAs are relatively stable in biological fluids such as blood.^10^ Blood is the most commonly used and sensitive specimen reflecting the health status of the human body and easy to obtain relative to other tissue samples used in studies of childhood diseases. Several miRNAs exhibited the potential to be biomarkers in patient blood, including miR-199a-3p,^11^ miR-27a-3p,^12^ and miR-134^13^ based on studies in adults with temporal lobe epilepsy. Unfortunately, there are fewer studies in children with epilepsy relative to studies in adults.

In the present study, we compared the miRNA profiles in blood plasma from pediatric patients with epilepsy and in healthy children using the miRNA-seq technique. The level of one identified miRNA candidate was further examined in additional patient plasma samples. The down stream target genes are examined in HEK293 cells and in epileptic mice. We hope that our results will be useful in identifying new diagnostic and therapeutic markers for epilepsy.

## Materials and methods

### Clinical sample acquisition and processing

According to the agreement approved by the Ethics Committee of the Second Affiliated Hospital of Wenzhou Medical University (#LCKY2020-12), we collected whole blood samples from children with new-onset epilepsy and healthy children. Peripheral blood (2 ml) was collected, and within 1 h the blood samples were centrifuged at 2000 rpm for 20 min at 4 °C to separate blood cells from plasma. The plasma was collected in a test tube without RNase and stored at −80 °C.

### MiRNA-seq and data analysis

The Illumina miRNA sequencing library was generated with TruSeq Small RNA Sample Prep Kits (Illumina, San Diego) and used for Illumina HiSeq 2000/2500 sequencing (LC Bio Technology Co., Ltd. Hangzhou, China). Clean reads were obtained after the raw data underwent quality controls. The remaining sequences were compared with various RNA databases that contained no miRNAs. The final data obtained were then utilized for subsequent miRNA data analysis with the ACGT101-miR program (LC Sciences, Houston, TX). Differential expression of miRNAs was analyzed by selectively using Fisher’s exact probability test, a Chi-squared 2 × 2 contingency table, Chi-squared *n* × *n* test, Student’s *t* test, or ANOVA based on specific experimental design. The significance thresholds were set at 0.01 and 0.05 for each test.

### Cell culture and transient transfection

HEK-293T cells were purchased from Procell Life Science & Technology Co., Ltd. (Wuhan, China). Cells were transfected using RNATransMate (Sangon Biotech, Shanghai, China) with miR-27a-3p mimics (UUCACAGUGGCUAAGUUCCG), miR-27a-3p inhibitor (CGGAACUUAGCCACUGUGAA), and their negative controls: miR-27a-3p mimics negative control—control 1 (UUGUACUACACAAAAGUACUG) and miR-27a-3p inhibitor negative control—control 2 (CAGUACUUUUGUGUAGUACAA), which were ordered from Sangon Biotech (Shanghai, China).

### mRNA-seq and data analysis

Total RNA was extracted from cells using RNAiso Plus (Takara, Japan), and whole-genome transcriptome analysis was conducted (LC Bio Technology Co., Ltd. Hangzhou, China). A library with a fragment size of 300 ± 50 bp was generated and sequenced using Illumina NovaSeq sequence 6000 (LC Bio Technology Co., Ltd. Hangzhou, China). For RNA-seq data, fastp was used to control the original data, including removing connectors, repetitive, and low-quality sequences. HISAT2 was adopted to compare the sequencing data to the genome (*Homo sapiens*, GRCh38). The genes were assembled by StringTie and quantified by FPKM. The differentially expressed mRNAs were selected with a fold-change >2 or fold-change <0.5, and with the parametric F-test that compared nested linear models (*p* value < 0.05) using the R package edgeR (https://bioconductor.org/packages/release/bioc/html/edgeR.html).

### Western blot analysis

Equal amounts of proteins were separated by electrophoresis on a sodium dodecyl sulfate–polyacrylamide gel and transferred to a polyvinylidene fluoride membrane (PR05509, Millipore, Cork, Ireland). Antibodies included GOLM1 (1:4000; Proteintech, Cat# 15126-1-AP), LIMK1 (1:1000; affinity, Cat#AF6345), CBX1 (1:1000; Proteintech, Cat#10241-2-AP), and GAPDH (1:5000; Proteintech, Cat# 60004-1-Ig). Secondary antibodies were goat anti-rabbit IgG (1:5000, Abcam, Cat# AB97051) or goat anti-mouse IgG (1:5000, Abcam, Cat# AB97023). ChemiDox XRS (Bio-Rad) was used for detection, and pictures were analyzed with ImageJ software (NIH, Bethesda, MD).

### Epilepsy mouse model

C57BL/6 mice (6–8 weeks of age, 18–25 g) were used in this study. Animals were purchased from the Wenzhou Medical University Animal Experiment Center, Wenzhou, China (License No. SCXK [Zhe] 2018-228), and approved by the Experimental Animal Ethics Committee of Wenzhou Medical University (#SYXK2015-0009). Mice were randomly allocated to a control group (*n* = 6) and an epilepsy group (*n* = 14). Mice in the epilepsy group were intraperitoneally injected with lithium chloride (127 mg/kg; L812571, Macklin, Shanghai, China), and 15 h later with scopolamine (1 mg/kg; S0231, TCI, Shanghai, China). Thirty min later, pilocarpine (200 mg/kg; P344660, Aladdin, Shanghai, China) was injected intraperitoneally to induce seizures. Mice in the control group were injected with the same volume of normal saline. Seizure severity was rated using the Racine scale: category 1 reflected immobility and facial twitch; category 2, head nodding; category 3, forelimb clonus; category 4, rearing; and category 5, rearing and falling. Animals were excluded from the study if they did not develop a category 4–5 episode within 30 min of pilocarpine injection. The seizure was terminated by intraperitoneal injection of pentobarbital (10 mg/kg) 90 min after pilocarpine injection.

### Reverse transcription-quantitative PCR (RT-qPCR)

A miRcute Serum/Plasma miRNA Isolation Kit (TIANGEN, DP503, Beijing, China) was utilized to isolate miRNA from plasma, and a MiPure Cell/Tissue miRNA Kit (Cat. #RC201-BOX 2, Vayzme, Shanghai, China) was adopted to isolate miRNAs from mouse tissues and HEK-293T cells. A miRcute Plus miRNA First-Strand cDNA Kit (TIANGEN, KR211) and PCR thermal cycler (T100 Thermal Cycler, Bio-Rad, Singapore) were implemented to synthesize cDNA. RT-qPCR was performed on a CFX96 Real-Time PCR Assay System (Bio-Rad Laboratories, Inc., Hercules, CA) using the miRcute Plus miRNA qPCR Kits (TIANGEN, FP411). The reaction procedure was: 95 °C, 15 min; Cycle 1 (5 times): 94 °C, 20 s; 64 °C, 30 s; 72 °C, 34 s; Cycle 2 (44 times): 94 °C, 20 s; 60 °C, 34 s. An external reference (#CR100-01, TIANGEN, Beijing, China) was used in our RT-PCR to examine plasma samples, and U6 was used as an internal control for other RT-PCR experiments. Universal U6 primer (#B661602-0002) was purchased from Sangon Biotech Co. (Shanghai, China). The miR-27a-3p primer sequence was TATACGCTTCACAGTGGCTAAGTTCCG. Fold change calculations for miRNA expression were performed using the the 2^−ΔΔCt^ method.^14,15^ U6 served as an internal reference. For other RT-PCR amplifications, total RNA was isolated using an RNA extraction kit (R0026, Beyotime, Shanghai, China), and RT-qPCR was conducted using a TOROGreen qPCR Master Mix (QST-100, TOROIVD, Shanghai, China). The reaction procedure was as follows: 95 °C for 1 min; 40cycles: 95 °C 15 s; 60 °C, 15 s; 72 °C, 45 s. β-Actin was used as the internal control. The primers for GOLM1 (human): 5’-CGCCGTGGAGCTGAAGAAGAAC-3’ (F) and 5’-GCTGGAAGTTGTGGCTGGACTG-3’ (R); LIMK1 (human): 5’-AAGAATGTGGTGGTGGCTGACTTC-3’(F) and 5’-CTTGCGGTCTGGCTTCTTGAGG-3’(R); CBX1 (human), 5’-GAGGAGGTGCTAGAAGAGGAGGAAG-3’(F) and 5’-CTCCACTTTGCCCTTTACCACTCG-3’(R); GOLM1 (mouse), 5’-AGCCAGATGACCGAGGTGAAGG-3’(F) and 5’-CCTGCTGATGTTGGTTGTTGGTTTC-3’(R); and LIMK1 (mouse), 5’-CAGGCGAGGTGATGGTGATGAAG-3’(F) and 5’-GTCCTTGTAGAGCACTCCGATGAAC-3’(R).

### Tissue immunofluorescence staining

After inhalation of isoflurane anesthesia, the mouse brains were perfused with phosphate-buffered saline (PBS, P1020, Solarbio), fixed with paraformaldehyde (P1110, Solarbio), and dehydrated with sucrose; frozen sections were then cut at 24 μm on a computerized microtome (KD-3390, KEDEE, Jinhua, Zhejiang, China). Brain slices were permeabilized with Triton X-100 (0.2%) for 15 min, blocked in PBS containing 10% goat serum (SL038, Solarbio) for 1 h, and incubated with primary antibody overnight at 4 °C, followed by secondary antibody donkey anti-rabbit IgG (1:200; Cat# ab150074, AB_2636997, Abcam, Cambridge, UK) at room temperature for 1 h. We used 4’,6-diamidino-2-phenylindole (DAPI) anti-fluorescence attenuation mounting tablets (Solarbio, S2110) to mount the tissues, and photomicrographs were taken under a fluorescence microscope (Nikon TI-DH, Tokyo, Japan). The primary antibodies used were GOLM1 (1:200; Proteintech, Cat# 15126-1-AP), LIMK1 (1:100; affinity, Cat#AF6345), and Phospho-LIMK1 (1:200, affinity; Cat#AF3345). The mean fluorescence intensities of the immunofluorescence images were analyzed with ImageJ software.

### Statistical analysis

We did not apply statistical methods to predetermine sample sizes for this study. Animals that did not reach an appropriate level of seizure activity were euthanized and not included in the statistical sample data. The evaluator was blinded to the animal groupings, and all data were derived from at least three independent experiments. Data are presented as the mean ± the standard error of mean (SEM) or standard deviation (SD) where noted, and we implemented statistical analyses with GraphPad Prism 8.0 (GraphPad Software, San Diego, CA). Student’s *t* test was used for the comparison of the two groups, and probability values of *p* < 0.05 were considered to be statistically significant.

## Results

### MiRNA expression profiling is different in epileptic pediatric patients

Fourteen children included in our patient group were admitted to our hospital because they experienced their first seizure; their ages were from 5 months to 14 years, and the male-to-female ratio was 1:1. The seizure types were as follows: eight were focal, three were generalized, and three were categorized as spasm; 12 of the patients had abnormal EEGs. The 14 in the control group were healthy children who attended our hospital for regular physical examinations. The controls were from 10 months to 10 years of age, and their male-to-female sex ratio was 11:3. Other patient characteristics are depicted in Tables 1 and 2.

**Table 1:** Information on patients included in the study.

Characteristics	Patients	Controls
Sex	7 (male) 7 (female)	11 (male) 3 (female)
Age (months) Median and IQR (25% to 75% percentiles)	15 (5–72)	78 (45–96)
Seizure type	6 partial 5 generalized 3 spasm	N/A
Seizure frequency (times) (within 24 h) (mean ± SD)	4.1 ± 2.8	N/A
Seizure duration (s) Median and IQR (25% to 75% percentiles)	60 (27-570)	N/A
EEG	10 normal	N/A
	4 abnormal

**Table 2:** Additional information on participants from the patient and healthy control groups included in the study.

Patient	Sex	Age	Diagnosis	Seizure type	Seizure frequency (within 24 h)	Seizure duration	Time from sampling to seizure	EEG	Medication before blood sampling	Healthy control	Sex	Age
*E-1*	M	5 m	Epilepsy, infantile spasms	Spasm	5–6 times	2–10 min	1 h	Hypsarrhythmia	Chloral hydrate	*C-1*	M	6 y
*E-2*	F	14 m	Epilepsy	Generalized	5 times	3–5 min	1 h	Normal	Chloral hydrate	*C-2*	M	7 y
*E-3*	F	12 y	Epilepsy	Partial	5–6 times	1–2 min	1 h	Multifocal epileptic discharge	Carbamazepine	*C-3*	F	5 y
*E-4*	M	9 y	Epilepsy	Partial	5–6 times	1 min	4 h	Abnormal	Sodium valproate, Levetiracetam	*C-4*	F	8 y
*E-5*	F	5 m	Epilepsy	Generalized	2–3 times	0.5 min	1 h	Spike and slow wave emissions in the right occipital region	No medication	*C-5*	M	6 y
*E-6*	M	5 y	Epilepsy	Partial	1 time	30 min	1 h	Background rhythm slowed with no epileptic discharge	No medication	*C-6*	M	8 y
*E-7*	F	5 m	Epilepsy	Spasm	1 time	1 min	20 h	Hypsarrhythmia and seizures detected	No medication	*C-7*	M	8 y
*E-8*	M	15 m	Epilepsy	Spasm	3–4 times	Seconds	3 h	Spike and slow wave emissions in the central and parietal regions	No medication	*C-8*	M	7 y
*E-9*	M	14 y	Epilepsy	Partial	1 time	30 min	35 h	Spike and slow wave emissions in the central and parietal regions	No medication	*C-9*	M	10 y
*E-10*	M	5 m	Epilepsy	Partial	5–6 times	0.5–1 min	0.5 h	Epileptic discharge	Sodium valproate	*C-10*	M	9 y
*E-11*	M	3 y	Epilepsy	Partial	1 time	20 min	27 h	Epileptic discharge	No medication	*C-11*	M	3 y
*E-12*	F	15 m	Epilepsy	Partial	10 times	Seconds	1 h	Normal	No medication	*C-12*	F	10 m
*E-13*	F	15 m	Epilepsy	Generalized	3 times	1 min	1 h	Spike wave in the frontal area	Chloral hydrate	*C-13*	M	4 y
*E-14*	F	19 m	Epilepsy	Partial	8 times	1 min	1 h	Spike and slow wave emissions	Chloral hydrate	*C-14*	M	11 m

**Fig. 1:**
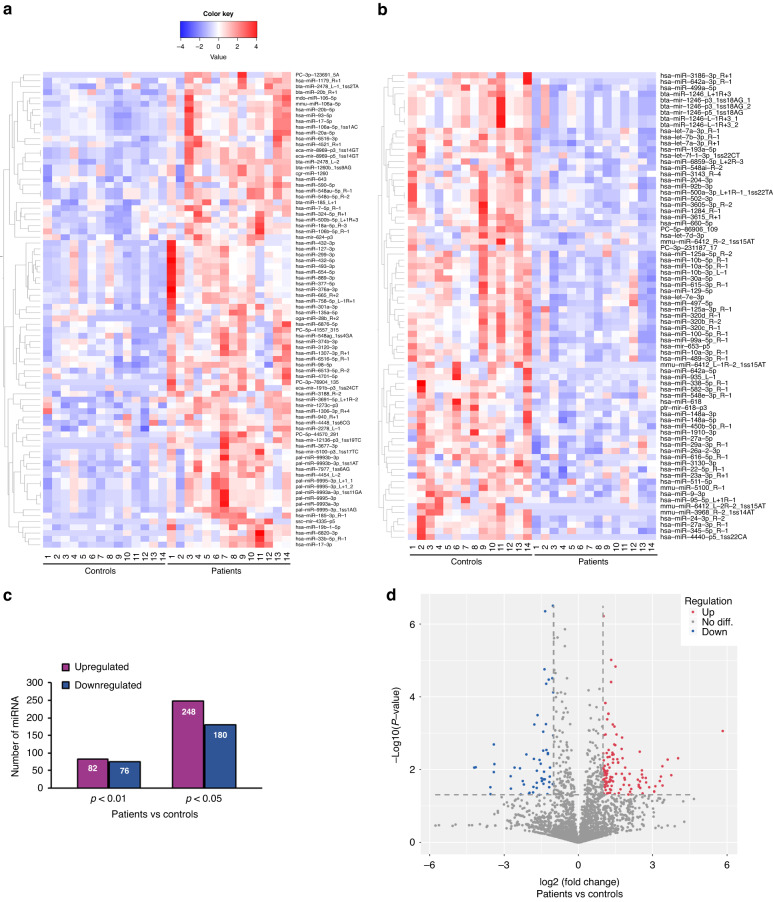
Results from miRNA-seq show significantly different (*p* < 0.01) miRNAs in plasma from the epilepsy group compared with the control group. **a** and **b**. Heatmap based on miRNA-seq results. Each row represents one miRNA, and each column represents one sample from the patient or control group. The names of the miRNAs are shown on the right side of the panel. The color scale at the top-left indicates the log2 fold-change from high (red) to low (blue). Upregulated genes are therefore represented in red and downregulated genes in blue. **a** Identified miRNAs that were upregulated in plasma from the patient group compared with the control group (*p* < 0.01). **b** miRNAs that were downregulated in plasma from the epileptic patient group compared with the controls (*p* < 0.01). **c** Histogram shows the number of differentially expressed miRNAs identified in plasma between the control and epileptic patient groups at *p* < 0.01 and *p* < 0.05. **d** Volcano plot shows the comparison of miRNA levels between the patient and control groups, where microRNAs with a fold-change >2 and *p* < 0.05 are shown as upregulated (red) or downregulated genes (blue), respectively.

We conducted miRNA-seq to reveal differences in miRNA levels between the plasma of the control group (*n* = 14) and the epilepsy group (*n* = 14) (the miRNAs with *p* values <0.01 are shown as heatmaps; Fig.1). There were 158 differentially expressed miRNAs with *p* values less than 0.01, of which 82 were upregulated (Fig. 1a, c) and 76 were downregulated (Fig. 1b, d). When our designated *p* value was <0.05 we noted 428 differentially expressed miRNAs, of which 248 miRNAs were upregulated and 180 were downregulated (Fig. 1c). When our restriction criteria were a fold-change of 2 combined with a *p* value < 0.05, our volcano plot delineated 102 miRNAs that were upregulated and 54 that were downregulated in patients compared with controls (Fig. 1d).

**Fig. 2:**
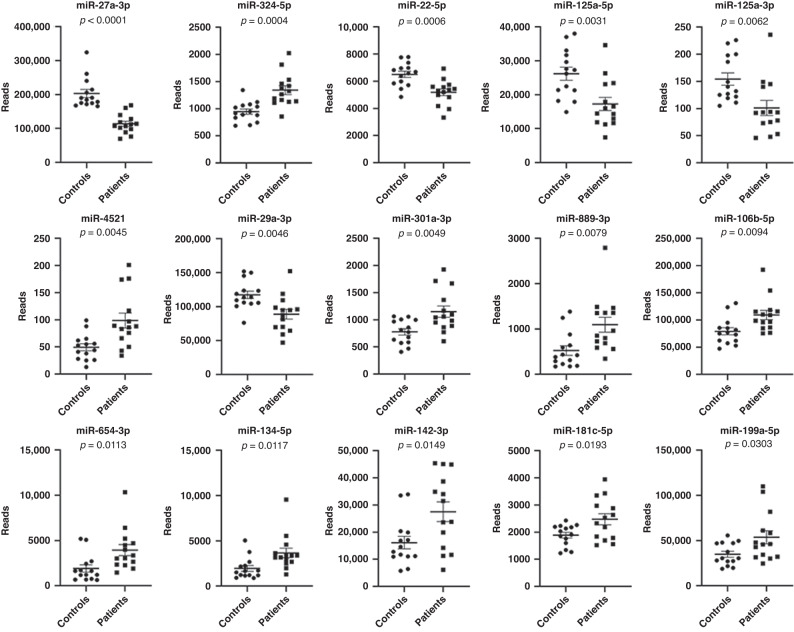
Scatter plots depict the miRNA-seq reads from our miRNA-seq screening, with *p* values listed above each scatter plot. The scatter plots illustrate the reads (mean with SEM provided) of miRNAs for each participant whose changing trend was consistent with the published literature, as shown in Table 3.

**Fig. 3:**
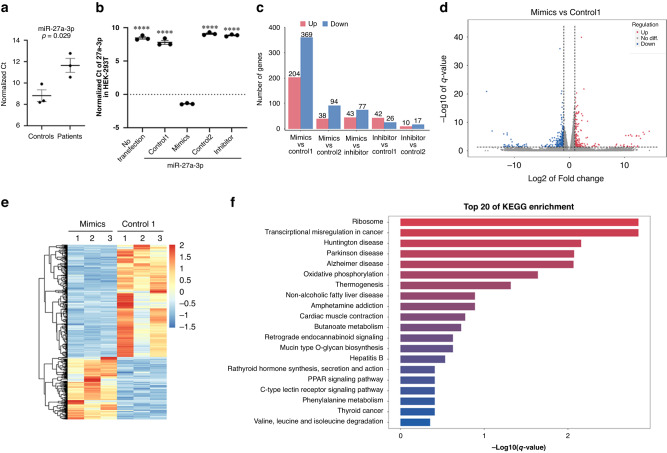
Using mRNA-seq to identify downstream targets of miR-27a-3p. **a** Validation of miR-27a-3p using RT-PCR in an expanded cohort that included controls (*n* = 3) and patients (*n* = 3). The *Y*-axis shows the normalized Ct value, with U6 as an internal reference. **b** The levels of miR-27a-3p as assessed with RT-PCR in HEK-293T cells with various transfections; the basal level of miR-27a-3p in HEK-293T cells is shown without transfection. MiR-27a-3p mimics significantly increased the levels of miR-27a-3p compared with all other groups. The MiR-27a-3p inhibitor did not decrease the levels of miR-27a-3p, suggesting that the basal level of miR-27a-3p in HEK-293T was already very low. **** represents p < 0.0001 from student ttest. **c** The histogram based on mRNA-seq results shows the number of differentially expressed genes (DEGs) between groups, with red indicating the number of upregulated genes (|log2FC| ≥ 1 and *q* < 0.05) and blue designating the number of genes that were downregulated (|log2FC| ≤ −1 and *q* < 0.05). **d** Volcano plot based on mRNA-seq results shows a comparison of gene levels between the miR-27a-3p mimics and the control 1, with log2FC as the abscissa, −log10 (*q*-value) as the ordinate, red representing upregulated significantly DEGs, and blue representing downregulated significantly DEGs genes. **e** The heatmap based on mRNA-seq results depicts a different expression profile between miR-27a-3p mimics and control, with the abscissa reflecting the sample and the ordinate reflecting the differentially expressed gene screened. **f** KEGG-enrichment bar plot based on mRNA-seq results comparing miR-27a-3p mimics and control 1. The Figure illustrates the top 20 KEGG pathways with the smallest *q*-value, the ordinate is the pathway name, and the abscissa is the −log10 value of the *q*-value for the GO item-enrichment analysis.

We compared miRNAs in the literature that were epilepsy related and those identified from patient blood with our miRNA profiling (Table 3). Fifteen miRNAs (*p* < 0.05) from our profiles illustrated the same changing trends as seen in the published reports (Table 3^12, 13,16–29^ and Fig. 2), and our data showed that miR-27a-3p was among the top-five miRNAs with the lowest *p* value when we compared the patient and control groups. Similar changes in miR-27a-3p were also reported in the plasma sequencing results of adult temporal lobe-epilepsy patients by Raoof et al.^12^. Although the epileptic conditions we applied for sequencing were not further classified, temporal lobe epilepsy (TLE) is the most common focal epilepsy in childhood, occurring in approximately 50–80% of focal cases.^30^ When we validated the miRNA-seq results of miR-27a-3p with RT-PCR using plasma from three additional children with epilepsy and three healthy controls (Fig. 3a), we observed that miR-27a-3p levels were significantly attenuated (*p* = 0.029, fold change = 0.27) in patients (the mean and SEM of CT is 11 ± 0.6) compared to controls (the mean and SEM of CT is 9 ± 0.5), similar change trend to the miRNA-seq results (Fig. 2). Although multiple authors suggest that the regulation of 27a-3p and its downstream target genes facilitate improvements in central nervous system diseases and produce brain-protective effects,^31–33^ the role and underlying mechanism(s) of action of miR-27a-3p in epilepsy remain largely unknown.

**Table 3:** miRNAs with different trends as observed from our miRNA-seq analysis (*p* < 0.05, average reads >50) are consistent with the published literature.

List	Name of miRNA	Our miRNA-seq result		Change in trend and description in references
		Change in trend	*p* value	
1	hsa-miR-27a-3p	Downward	<0.0001	Downregulation in plasma of patients with temporal lobe epilepsy^12^
2	hsa-miR-324-5p	Upward	0.000389	Upregulation in serum of children with epileptic encephalopathy^16^
3	hsa-miR-22-5p	Downward	0.000561	Downregulation in serum of mesial temporal lobe epilepsy^17^
4	hsa-miR-125a-5p/3p	Downward	0.003055/0.006221	Downregulation in plasma from children with epilepsy^18^
5	hsa-miR-4521	Upward	0.004530	Upregulation in serum and tissues from patients with focal cortical dysplasia with refractory epilepsy^19^
6	hsa-miR-29a-3p	Downward	0.004615	Downregulation in the serum of children with temporal lobe epilepsy^20^
7	hsa-miR-301a-3p	Upward	0.004947	Upregulation in serum of epilepsy patients^21^; upregulation in plasma and hippocampus of sudden and unexpected death in epilepsy (SUDEP) due to drug-resistant mesial temporal lobe epilepsy^22^
8	hsa-miR-889-3p	Upward	0.007872	Upregulation in serum of epilepsy patients^23^
9	hsa-miR-106b-5p	Upward	0.009361	Upregulation in serum of epilepsy patients^23^; upregulation in serum of epilepsy patients^21, 24^; and upregulated in plasma of children with epilepsy^25^
10	has-miR-654-3p	Upward	0.011322	Upregulation in epilepsy baseline-state plasma and seizure-state plasma^12^
11	hsa-miR-134-5p	Upward	0.011665	Upregulation in the plasma of epilepsy patients may be directly associated with the pathophysiology and severity of epilepsy,^13^ upregulation in plasma and cerebrospinal fluid of epilepsy patients^26, 27^
12	hsa-miR-142-3p	Upward	0.014943	Upregulation in the serum of temporal lobe epilepsy patients^28^
13	hsa-miR-181c-5p	Upward	0.019324	Upregulation in the blood of patients with mesial temporal lobe epilepsy with hippocampal sclerosis^29^
14	hsa-miR-199a-5p	Upward	0.030277	Upregulation in the blood of patients with mesial temporal lobe epilepsy with hippocampal sclerosis^29^

### Identification of the downstream target genes of miR-27a-3p via an unbiased mRNA-seq approach

Investigators have previously identified potential target genes of miR-27a-3p (Table 4^34–43^). To validate these downstream target genes and identify novel targets of miR-27a-3p, we exploited an unbiased mRNA-seq approach to achieve this goal. We transfected HEK-293T with specific miR-27a-3p mimics, inhibitors, and related negative controls, and we collected total RNA 24 h after transfection. mRNA-seq was then performed to reveal differentially expressed mRNAs among the four groups, and we analyzed miR-27a-3p in HEK-293T cells at basal levels (i.e., without transfection) and after various transfections (Fig. 3b). Although MiR-27a-3p mimics significantly augmented the levels of miR-27a-3p compared with all other groups, we observed no significant differences in miR-27a-3p levels between untransfected controls and transfection with miR-27a-3p inhibitor, suggesting that the basal levels of miR-27a-3p in HEK-293T were already exceedingly low. Indeed, mRNA-seq results showed that the differences between the MiR-27a-3p inhibitor group and controls were much smaller than the differences between the miR-27a-3p mimics and controls (Fig. 3c). We therefore subsequently focused our analysis on comparisons between miR-27a-3p mimics and controls (Fig. 3d–f). Based on KEGG pathway analysis, miR-27a-3p overexpression activated neuronal disease-related pathways such as Huntington’s disease, Parkinson’s disease, Alzheimer’s disease, oxidative phosphorylation, and thermogenesis pathway (Fig. 3f).

**Table 4:** Published target genes of miR-27a-3p that are also identified from our mRNA-seq screening (*p* < 0.05).

List	Gene ID	Gene names	References	Results from our screening (miR-27a-3p overexpression/control)		MiR-27a-3p-binding site (based on human gene sequence and prediction from the website at: https://www.targetscan.org/vert_80/)
				*p* value	Ratio	
1	ENSG00000106683	LIMK1	34	0.00007	0.414	Position 1057–1064 of LIMK1 3’ UTR
2	ENSG00000253293	HOXA10	35	0.0003	0.529	Position 1032–1039 of HOXA10 3’ UTR
3	ENSG00000065559	MAP2K4	36	0.001	0.749	Position 2178–2185 of MAP2K4 3’ UTR
4	ENSG00000248866	USP46	37	0.001	0.574	Position 1367–1374 of USP46 3’ UTR
5	ENSG00000266094	RASSF5	38	0.007	0.786	Position 2045–2051 of RASSF5 3’ UTR
6	ENSG00000109670	FBXW7	39	0.008	0.728	Position 503–510 of FBXW7 3’ UTR
7	ENSG00000177885	GRB2	40	0.011	0.823	Position 2260–2267 of GRB2 3’ UTR
8	ENSG00000104332	SFRP1	41	0.015	0.378	Position 3030–3037 of SFRP1 3’ UTR
9	ENSG00000106799	TGFβR1	42	0.037	0.786	Position 1617–1623 of TGFBR1 3’ UTR
10	ENSG00000140092	FBLN5	43	0.049	0.744	Position 342–349 of FBLN5 3’ UTR

**Fig. 4:**
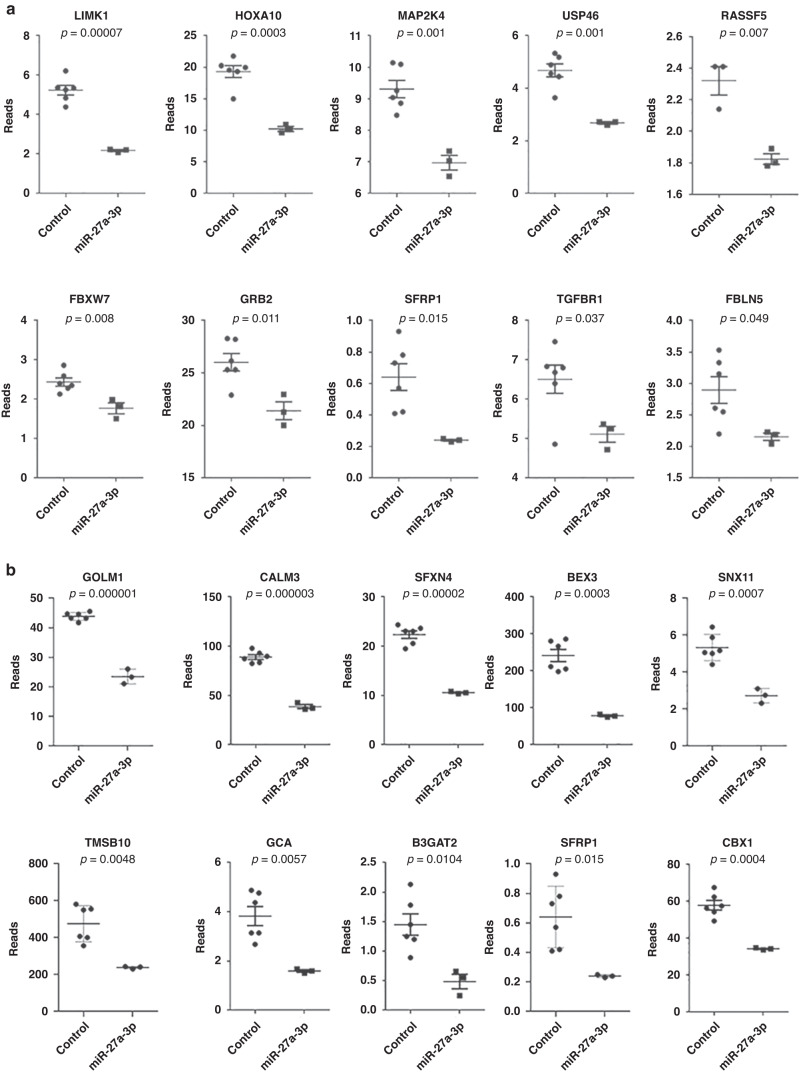
Scatter plot represents normalized reads (means with SEM are shown) of genes in our mRNA-seq library. **a** Genes published as regulated by miR-27a-3p, with specific *p* values listed above the scatter plot. **b** Genes with binding sites to miR-27a-3p in our RNA-seq library (unpublished), with specific *p* values listed above the scatter plot.

Because a majority of the direct effects of miRNAs on target genes reflected downregulation in our analysis, we then focused our attention on these downregulated genes after miR-27a-3p overexpression. We uncovered 219 protein-coding genes that were downregulated (*p* < 0.01) in the miR-27a-3p-mimics group compared with controls. Compared with reports in the literature, the published target genes of miR-27a-3p were also identified from mRNA-seq screening (*p* < 0.05) as shown in Table 4 and Fig. 4a. Potential miR-27a-3p-binding sites predicted using TargetScan (https://www.targetscan.org/vert_72/) on these genes were also listed. In addition, we showed another 10 novel target genes that were significantly (*p* ≤ 0.01) downregulated by miR-27a-3p overexpression (Table 5 and Fig. 4b).

**Table 5:** Novel identified target genes of miR-27a-3p from our mRNA-seq screening (*p* < 0.05).

List	Gene ID	Gene names	Results from our screening (miR-27a-3p overexpression/control)		MiR-27a-3p-binding site (based on human gene sequence and prediction from the website at: https://www.targetscan.org/vert_80/)
			*p* value	Ratio	
1	ENSG00000135052	GOLM1	0.000001	0.54	Position 79–86 of GOLM1 3’ UTR
2	ENSG00000160014	CALM3	0.000003	0.43	Position 1576–1582 of CALM3 3’ UTR
3	ENSG00000183605	SFXN4	0.00002	0.47	Position 271–278 of SFXN4 3’ UTR
4	ENSG00000166681	BEX3	0.0003	0.33	Position 27–33 and 91–97 of NGFRAP1 3’ UTR
5	ENSG00000108468	CBX1	0.0004	0.59	Position 1436–1442 of CBX1 3’ UTR
6	ENSG00000002919	SNX11	0.0007	0.51	Position 1826–1832 of SNX11 3’ UTR
7	ENSG00000034510	TMSB10	0.0048	0.50	Position 96–103 of TMSB10 3’ UTR
8	ENSG00000115271	GCA	0.0057	0.42	Position 29–36 of GCA 3’ UTR
9	ENSG00000112309	B3GAT2	0.0104	0.33	Position 1464–1470 of B3GAT2 3’ UTR
10	ENSG00000104332	SFRP1	0.015	0.38	Position 3030–3037 of SFRP1 3’ UTR

**Fig. 5:**
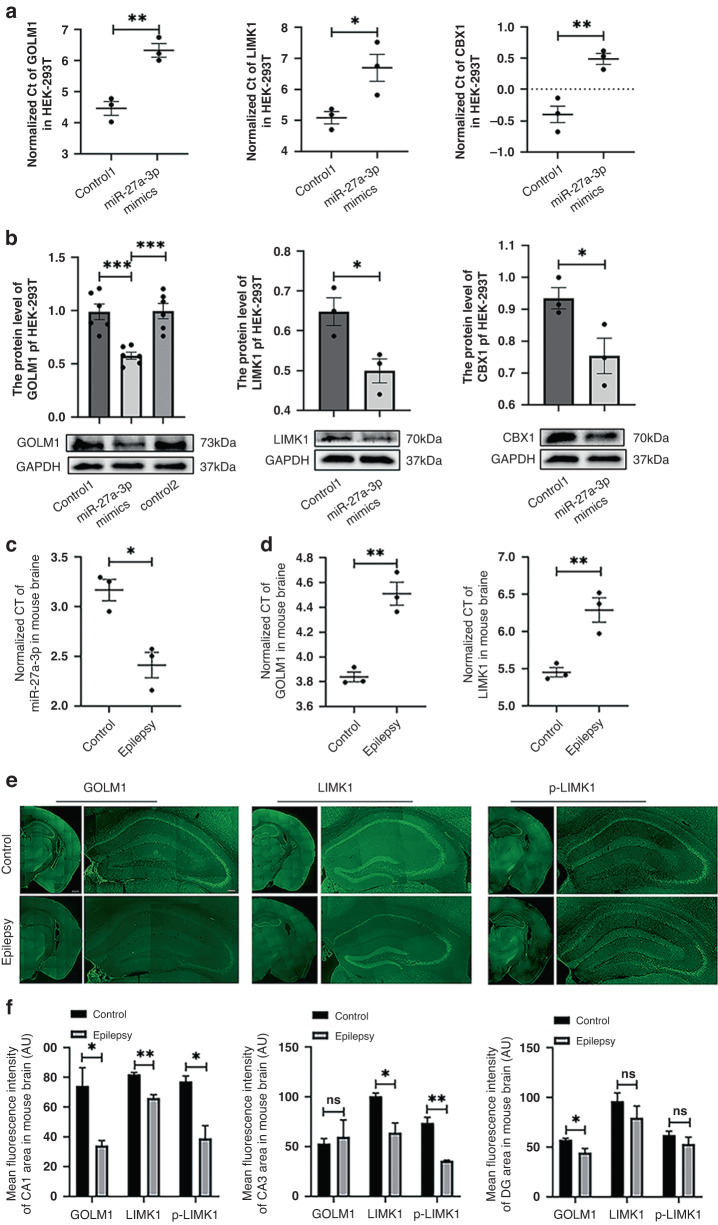
Changes in genes and proteins after overexpression of miR-27a-3p in HEK-293T cells (*n* = 3). Data are presented as mean ± SEM (*n* = 3), *p < 0.05, **p < 0.01, and ***p < 0.001 compared with the control group. **a** The mRNA expression levels of GOLM1, LIMK1, and CBX1 in HEK-293T cells after overexpression of miR-27a-3p, with β-actin used as an internal reference standard. **b** Quantitative analysis of GOLM1, LIMK1, and p-LIMK1 protein levels in HEK293 cells (normalized to GAPDH). Representative WB results are shown. **c** The expression levels of miR-27a-3p in mouse plasma were determined 1 h after epilepsy was terminated, with the external standard used as a reference control; the expression level of miR-27a-3p in mouse brain 6 h after epilepsy was terminated, with U6 used as a reference. The expression of miR-27a-3p in plasma and whole brain in the epilepsy group was upregulated. **d** RNA expression levels of GOLM1 and LIMK1 in the whole brain of mice 6 h after epilepsy was terminated (GAPDH was used as an internal reference for GOLM1 and LIMK1). **e** Representative immunofluorescence staining images of GOLM1, LIMK1, and P-LIMK1 (green) in mouse hippocampus 6 h after epilepsy termination. Staining levels of GOLM1, LIMK1, and P-LIMK1 were reduced in the epilepsy group (brain scale bars, 500 μm; hippocampal scale bars, 100 μm). **f** Quantification data of mean fluorescence intensity of mouse hippocampal CA1, CA3, DG region of GOLM1 (*n* = 3), LIMK1 (*n* = 3), and P-LIMK1 (*n* = 3). Data are presented as mean ± SEM; *p < 0.05 and **p < 0.01 vs. the control group.

To confirm the results from mRNA-seq, we chose LIMK1, GOLM1, and CBX1, as these genes showed the smallest *p* values (Tables 4 and 5). The mRNA and protein levels of LIMK1, GOLM1, and CBX1 are analyzed using RT-PCR, Western blot, and immunostaining. As shown in Fig. 5a, b, miR-27a-3p mimics significantly decreased the mRNA and protein levels of GOLM1, LIMK1, and CBX1 compared with the control.

### Levels of miR-27a-3p are increased in epileptic mice

To study the role of miR-27a-3p in epilepsy, we developed a mouse epileptic model induced by lithium chloride-pilocarpine. Previous studies revealed that miR-27a-3p was expressed in brain^12^ and that miRNA-27a levels in epileptic patient brain tissues were elevated relative to controls.^44, 45^ We therefore assessed the levels of miR-27a-3p in the mouse brain after epilepsy induction using RT-PCR and found that the levels were significantly higher in the epilepsy group compared with the control group (Fig. 5c); this tendency was also the same for patient brains.^44,45^ Previous reports using the epileptic rat model showed significantly higher expression of miR-27a-3p in the brains of epileptic rats,^45,46^ and we also discerned that the mRNA levels of GOLM1 and LIMK1 were significantly lower in the brains of epileptic mice than in controls (Fig. 5d), consistent with miR-27a-3p effects on GOLM1 and LIMK1 in HEK293T cells.

By immunofluorescence staining, we ultimately examined the protein levels of GOLM1, LIMK1, and P-LIMK1 (the active form of LIMK1) in the brains of epileptic mice compared with controls (Fig. 5e). The hippocampal region of the brain is well known for its strong association with epilepsy, and in the epileptic mouse brain the levels of GOLM1 were significantly attenuated in hippocampal CA1 and dentate gyrus areas, while LIMK1 and P-LIMK1 levels were significantly reduced in the hippocampal CA1 and CA3 areas compared with controls (*p* < 0.05, Fig. 5f). Thus, GOLM1 and LIMK1 protein levels were diminished in the mouse hippocampus, while miR-27a-3p levels in the mouse brain were increased after seizure induction.

## Discussion

In this study, we compared the miRNA profiles in blood plasma from pediatric epilepsy patients with those from healthy children using miRNA-seq and identified levels of 158 miRNAs that were significantly different between the two groups. The *p* value of miR-27a-3p between patients and controls was one of the lowest, and further verified in additional plasma samples using RT-PCR. Using unbiased mRNA-seq, we compared the mRNA profiles in HEK-293T cells that overexpressed miR-27a-3p with control HEK-293T cells to identify downstream targets of miR-27a-3p, and we uncovered 219 protein-coding genes that were downregulated (*p* < 0.01). Two genes—GOLM1 and LIMK1—were further validated in both HEK293T cells and epileptic mice using WB, RT-PCR, and immunostaining.

Numerous studies have revealed that miRNAs acting as critical post-transcriptional gene regulators are involved in various diseases. Because studies on epilepsy patients showed many dysregulated miRNAs in the brain or circulating fluids, miRNAs have the potential to develop into diagnostic biomarkers or therapeutic targets. In our study, 15 miRNAs identified from our miRNA-seq analysis manifested the same tendency with previous reports. Among these miRNAs, miR-27a-3p levels in 13 of the 14 patient samples were all lower than in the 14 control samples. Raoof et al. reported that miR-27a-3p levels were also lower in the blood plasma of epilepsy patients whose blood samples were collected after an electro-clinical seizure than in controls, and their data on miR-654-3p were consistent with our results.^12^ Our mean level of miRNA miR-328-3p (which was also reported in their study) was lower in our patient group but not to a statistical degree. These authors also demonstrated that miR-27a-3p levels were significantly lower in plasma samples from epileptic mice than in controls.^12^ In both the Raoof et al.^12^ and our studies, the blood samples were collected from patients after experiencing a seizure. In another study, the levels of miR-27a-3p were not significantly changed in the blood samples collected from patients with mesial temporal lobe epilepsy with hippocampal sclerosis (MTLE-HS) who underwent anterior temporal lobectomy + amygdalohippocampectomy 18 months previously.^47^ However, the authors did not mention the epilepsy status of the patients when the blood samples were collected.

Although an association between epilepsy and dysregulation of miRNAs has been suggested by many studies, the subserving mechanism is still not fully understood. miR-27a-3p plays roles in neurological disorders, including regulations of cerebral ischemia/reperfusion injury,^33,48^ cerebral endothelial barrier permeability,^49,50^ inflammatory response, and apoptosis of hippocampal neurons in epilepsy.^46^ We examined miR-27a-3p and its downstream targets in the brains removed from the lithium chlorine-pilocarpine-induced epilepsy mouse model, and we noted that the levels were elevated, consistent with other reports using epilepsy animal models.^45,46,51,52^ Importantly, miR-27a-3p levels were also higher in the brain tissues from epilepsy patients than in controls.^44,45,53^

Because the direct actions of miRNAs occur via binding to the 3’UTR of target mRNA to inhibit their expression, we used unbiased mRNA-seq analysis to identify target genes of miR-27a-3p. We identified 10 genes reported previously, compared miR-27a-3p overexpression and controls (*p* < 0.05), and uncovered another 10 potential target genes with a miR-27a-3p functional response element on their 3’UTR regions. Accordingly, the protein levels of two miR-27a-3p target genes, GOLM1 and LIMK1, were found to drop in the hippocampal regions of the epileptic mouse brain while miR-27a-3p levels remained elevated.

GOLM1 (also termed GP73 or GOLPH2) is a type II transmembrane protein residing on the cis and medial regions of the Golgi apparatus that can be secreted into blood.^54^ GOLM1 is significantly upregulated in various types of cancers such as hepatocellular carcinoma, non-small cell lung cancer, cutaneous melanoma, cerebroma, and glioblastoma multiforme.^55^ Because it is also secreted into the blood, GOLM1 is regarded as a novel serum biomarker for the diagnosis of disparate cancers. Polymorphisms rs10868366 and rs7019241 in GOLM1 were identified as Alzheimer’s disease risk factors,^56,57^ suggesting potential functions of GOLM1 in neurons. Further analysis study is required to elucidate GOLM1’s role in brain diseases that include epilepsy.

Consistent with a previous report,^34^ our results showed that miR-27a-3p mimics diminish the levels of LIMK1, suggesting that LIMK1 is a direct target of miR-27a-3p. LIMK1 encodes a cytoplasmic serine/threonine kinase expressed in neurons of the central nervous system, with phosphorylated LIMK1 (p-LIMK1) as the active form of the molecule. LIMK1 is known to be the principal target of another microRNA, miR-134, which is well known for its robust potential as a diagnostic biomarker and therapeutic target in epilepsy.^58^ As a brain-enriched miRNA, the levels of miR-134 are augmented in the blood of epilepsy patients,^13,26,27^ consistent with our miRNA-seq results. MiR-134 levels also rise in epileptic brains from both patients and multiple epileptic animal models.^27,59^ If both miR-134 and miR-27a-3p levels are high in epileptic brains, then LIMK1 protein levels should drop; indeed, both our results and reports in the literature confirm this hypothesis.^60,61^ LIMK1 is an important regulator of actin and microtubule dynamics through the ROCK1/LIMK1/COFILIN1-signaling pathway.^62–64^ In the nervous system, LIMK1 is crucial in increasing the number of dendritic spines, stimulating the growth of axons and dendrites,^65^ and maintaining mature synapses.^66^ Regarding the relationship between cytoskeletal dynamics and epilepsy, it was reported that actin and the cytoskeleton in general were deconstructed in the hippocampus after epileptic seizure to regulate high-dynamic microtubules, and this appeared to produce anti-epileptic effects.^67^ Dysfunction of the microtubular cytoskeleton may therefore predispose nerves to abnormal neurotransmission^68,69^ as the neuronal tubule cytoskeleton also portrays an important role in regulating voltage-gated ion channel activity and the affinity of several neurotransmitters for their receptors.^70^ Therefore, both miR-27a-3p and miR-134 may regulate cytoskeletal structure through LIMK1 to be involved in epilepsy. Unraveling the potential interplay between miR-27a-3p and miR-134 necessitates additional study.

There are many limitations to consider in interpretation of our study. We do not know how generalizable our findings are since the patients were enrolled within a limited time period and the group sizes for both patients and controls are small. The median age (15-month) of the patient group is much younger than that (78-month) of the healthy control group. Age differences alone could influence the miRNA profiles in blood samples. Meanwhile, the sex ratios in patient group (7 M:7 F) is different from the control group (11 M:3 F), which can also affect the miRNA profiles in blood samples. Our use of a single time point for blood sampling is restrictive and we did not adjust for time-of-day sampling which could affect miRNA profiles too. Therefore, it will be more informative or accurate to include serial sampling in the future. Another limitation is that only one internal control was used to normalize miRNA-27a-3p levels examined by RT-PCR. Many studies are using several internal control genes or standard housekeeping genes in RT-PCR tests to normalize the target readings and enable more accurate results. In addition, the evidence is limited that circulating miRNAs in patients with epilepsy actually come from the brain. Our examination of miR-27a-3p in the brain of mouse model does not directly explain its levels in the blood. Future experiments should focus on identifying the cellular origins of circulating miRNAs in epilepsy models. More independent validations of findings are needed from other teams to increase confidence in these findings.

## Conclusions

MiR-27a-3p exhibits potential as a diagnostic and therapeutic marker for epilepsy. MiR-27a-3p acts through its downstream target genes such as GOLM1 and LIMK1, or cooperates with other miRNAs such as miR-134, to allow its participation in brain functions. We postulate that additional studies on the downstream targets of miR-27a-3p will unravel its roles in epileptogenesis or disease progression.

## Data Availability

The datasets used and/or analyzed during the current study are available from the corresponding author upon reasonable request.
